# Supplementary material for: Quantification of cytosolic interactions identifies Ede1 oligomers as key organizers of endocytosis
Source: Mol Syst Biol. 2014 Nov 3;10(11):756. doi: 10.15252/msb.20145422 (PMC4299599; doi:10.15252/msb.20145422)
Supplement: Supplementary file 1 — Supplementary Figure S1 [file msb0010-0756-sd1.pdf]

Figure S1

Boeke et al. 2014

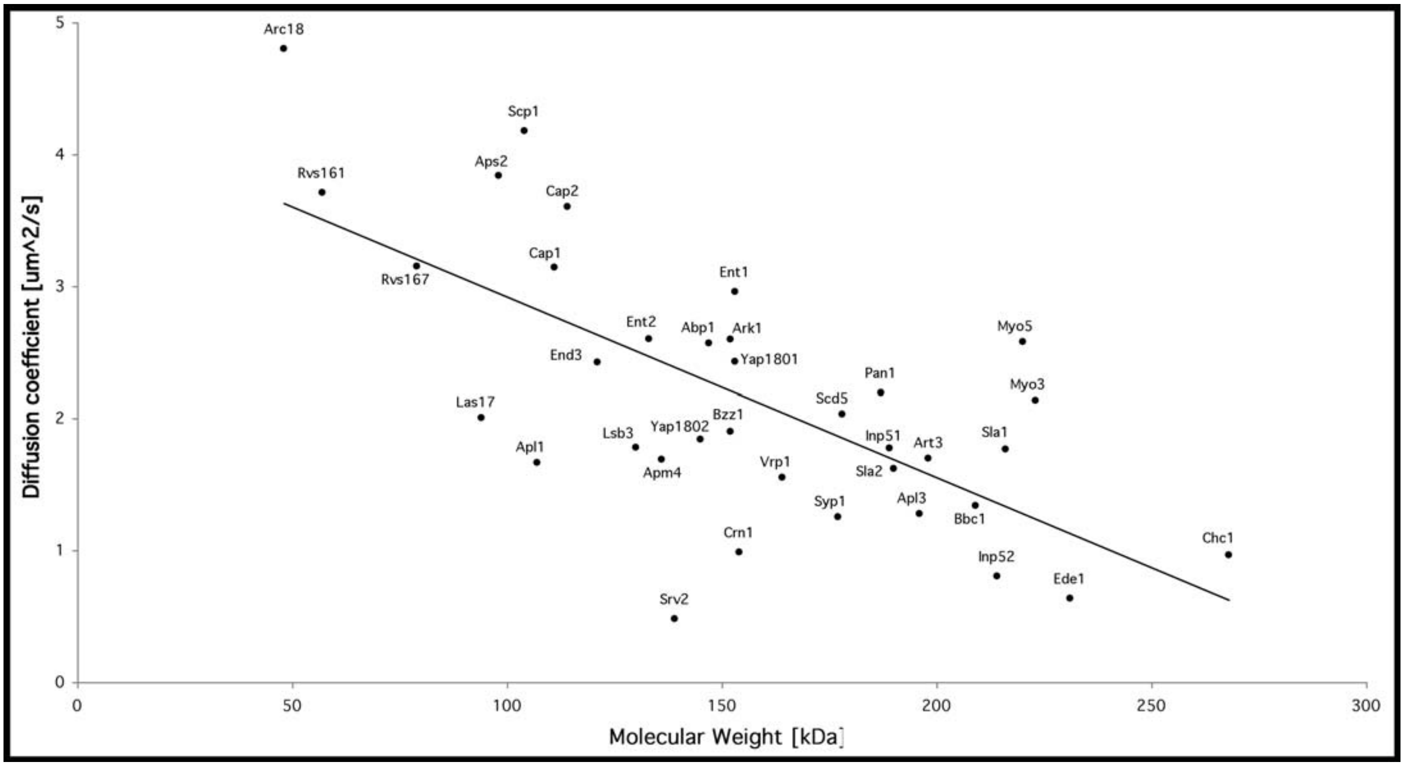

Figure S1. Diffusion coefficient measured by FCS of the indicated proteins plotted as a function of molecular weights, considering the tagged variants of the proteins.
