# Supplementary material for: Quantification of cytosolic interactions identifies Ede1 oligomers as key organizers of endocytosis
Source: Mol Syst Biol. 2014 Nov 3;10(11):756. doi: 10.15252/msb.20145422 (PMC4299599; doi:10.15252/msb.20145422)
Supplement: Supplementary file 2 — Supplementary Figure S2 [file msb0010-0756-sd2.pdf]

Figure S2

Boeke et al. 2014

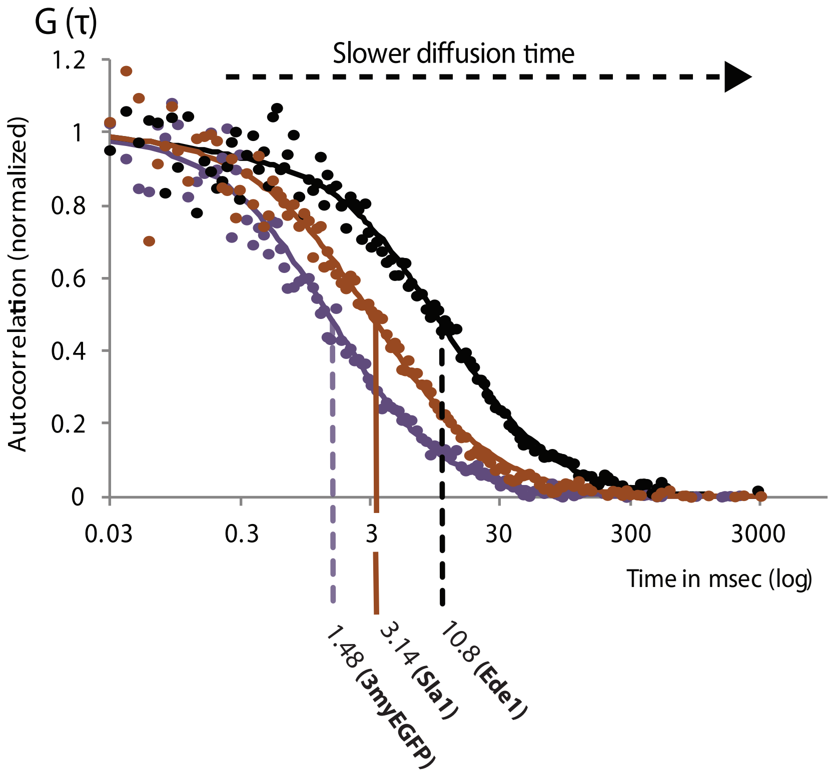

Figure S2. Example of a fitted FCS trace for 3myeGFP (purple), Sla1 (brown) and Ede1 (black) with the determined average diffusion time of the particles. Please note that diffusion time is inversely proportional to diffusion coefficient. Correlation curves were normalized for better visualization of the diffusion behavior.
