# Supplementary material for: Quantification of cytosolic interactions identifies Ede1 oligomers as key organizers of endocytosis
Source: Mol Syst Biol. 2014 Nov 3;10(11):756. doi: 10.15252/msb.20145422 (PMC4299599; doi:10.15252/msb.20145422)
Supplement: Supplementary file 3 — Supplementary Figure S3 [file msb0010-0756-sd3.pdf]

Figure S3

Boeke et al. 2014

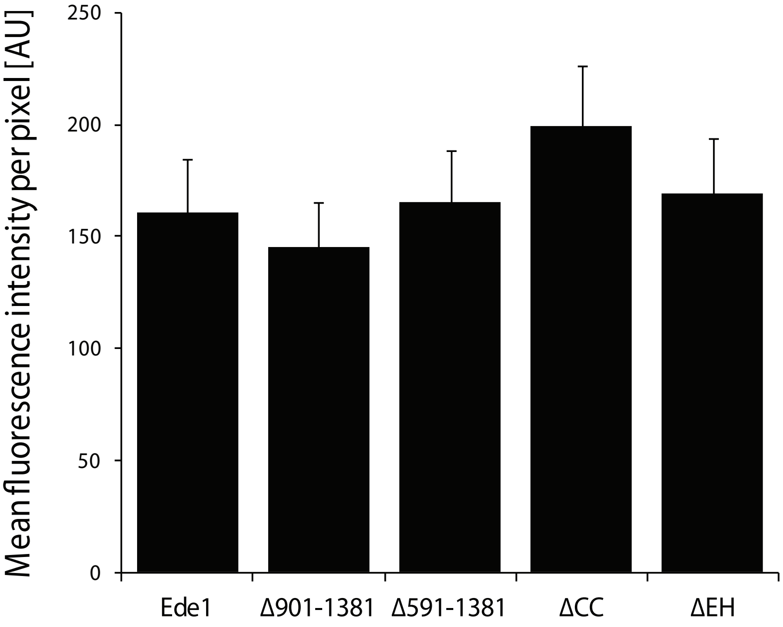

Figure S3. Mean Fluorescent intensity per pixel in whole cells harbouring Ede1-1myEGFP and different ede1 mutants tagged with 1myEGFP. At least 22 cells were measured for each strain. Error bars represent standard deviation.
