# Supplementary material for: Quantification of cytosolic interactions identifies Ede1 oligomers as key organizers of endocytosis
Source: Mol Syst Biol. 2014 Nov 3;10(11):756. doi: 10.15252/msb.20145422 (PMC4299599; doi:10.15252/msb.20145422)
Supplement: Supplementary file 4 — Supplementary Figure S4 [file msb0010-0756-sd4.pdf]

Figure S4

Boeke et al. 2014

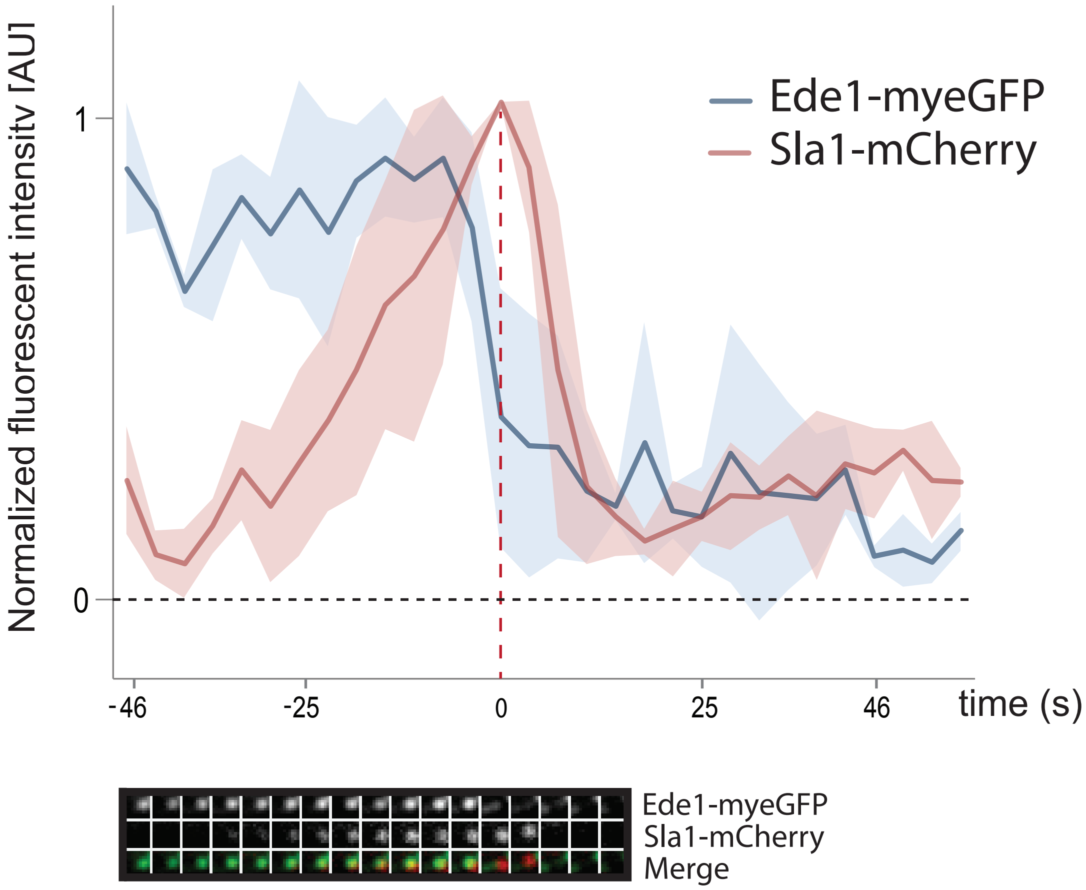

Time interval 3.5 sec

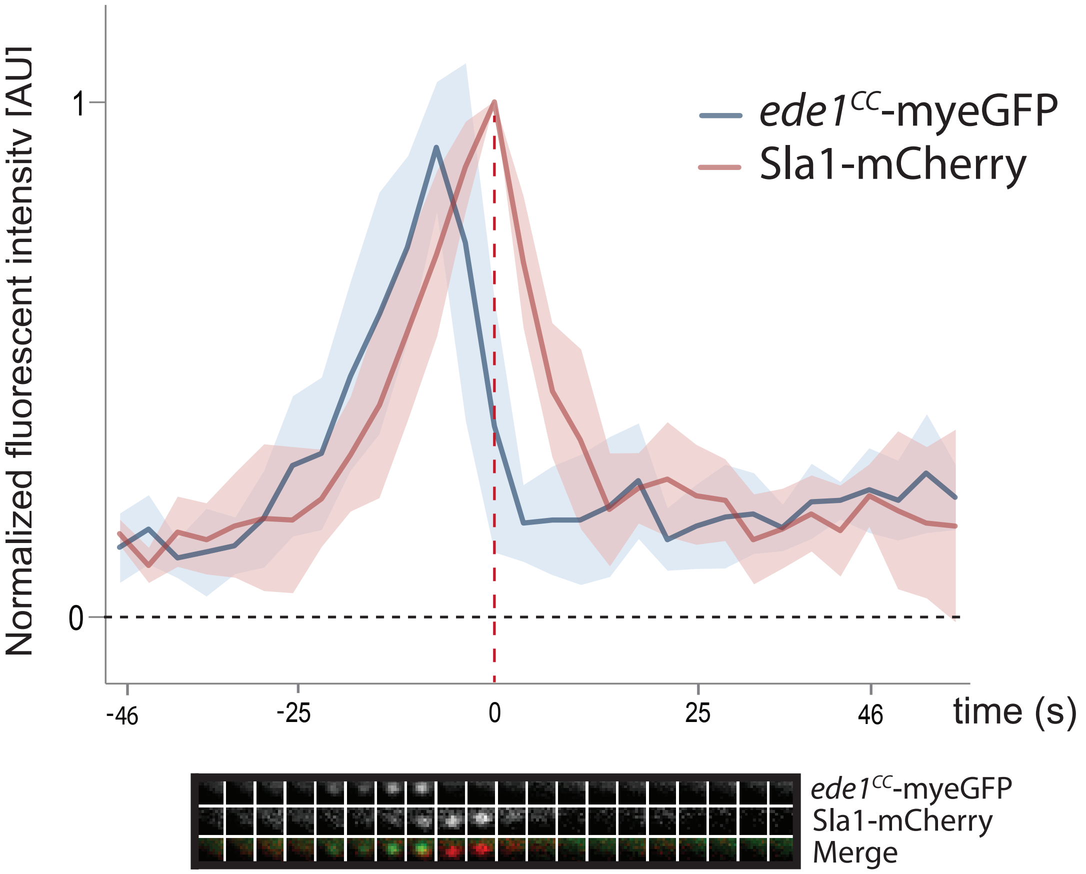

Time interval 3.5 sec

Figure S4. Quantification of the fluorescent intensity for Ede1-myeGFP/Sla1-mCherry patches (N=4, top panel) or *ede1cc*-myeGFP/Sla1-mCherry patches (N=8, bottom panel) as a function of time. Individual intensity curves for GFP (blue) and mCherry (red) were normalized independently. For each patch, the GFP and mCherry curves were aligned to the peak intensity of Sla1 (= time point 0) in time. Below each graph, a time series of a representative patch is shown, that has been adjusted independently for better visualization.
