# Supplementary material for: Quantification of cytosolic interactions identifies Ede1 oligomers as key organizers of endocytosis
Source: Mol Syst Biol. 2014 Nov 3;10(11):756. doi: 10.15252/msb.20145422 (PMC4299599; doi:10.15252/msb.20145422)
Supplement: Supplementary file 5 — Supplementary Figure S5 [file msb0010-0756-sd5.pdf]

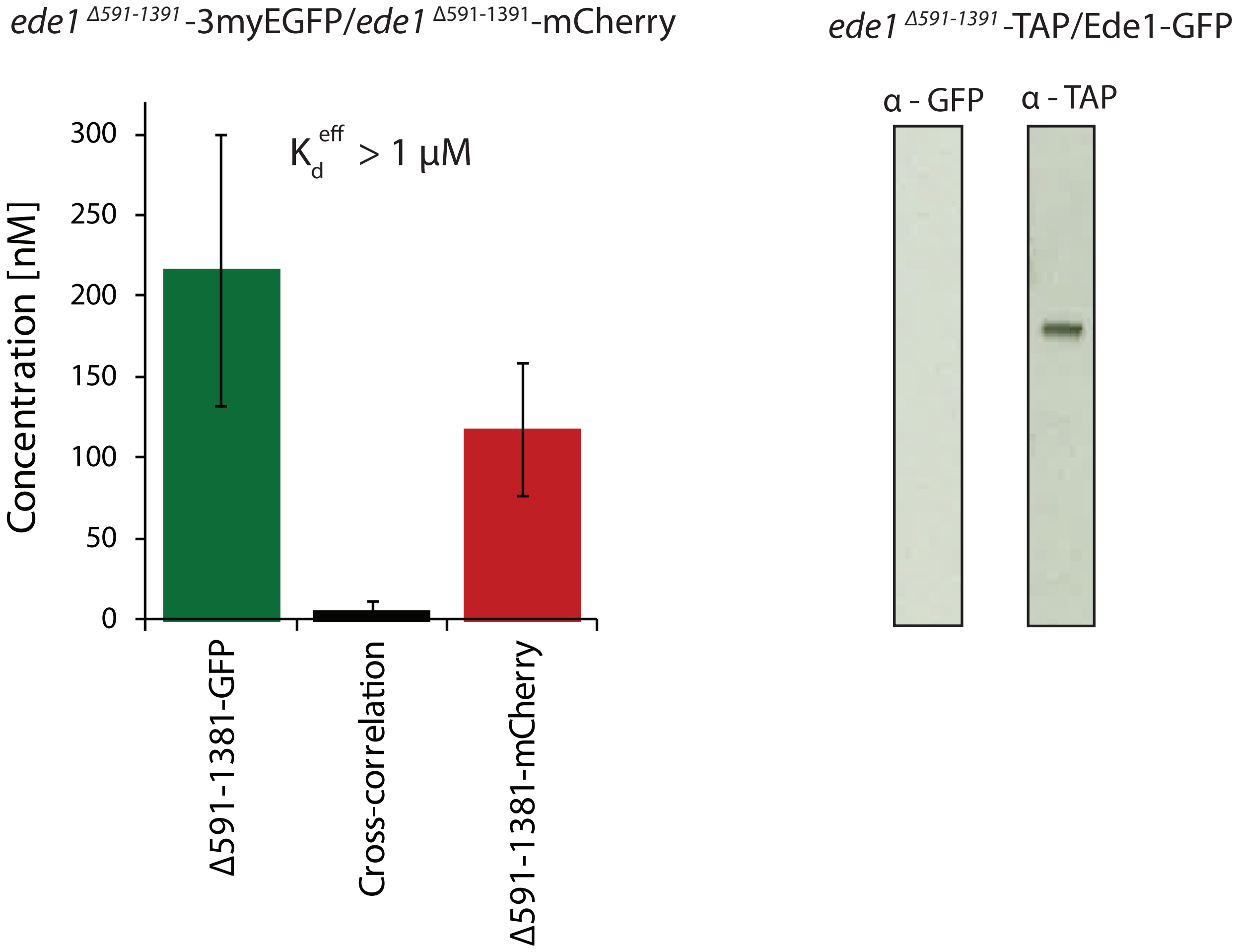
ede1 Δ591-1391-3myEGFP/ede1 Δ591-1391-mCherry
K_d^{eff} > 1 µM
Concentration [nM]
300
250
200
150
100
50
0
Δ591-1381-GFP
Cross-correlation
Δ591-1381-mCherry
ede1 Δ591-1391-TAP/Ede1-GFP
α - GFP
α - TAP

Figure S5. Left panel: Tandem affinity purification of a diploids *ede1*$_{\Delta 591\text{-}1391}$-TAP/Ede1-eGFP strain. Purified proteins were subject to SDS-PAGE and were immunoblotted with α-GFP to detect Ede1-eGFP and α-TAP to detect Ede1-TAP. Right panel: FCCS measurements of *ede1*$_{\Delta 591\text{-}1381}$-3myeGFP/ *ede1*$_{\Delta 591\text{-}1381}$-mCherry. Measurements were performed in 40 individual cells. Error bars represent standard deviation.
