# Supplementary material for: Quantification of cytosolic interactions identifies Ede1 oligomers as key organizers of endocytosis
Source: Mol Syst Biol. 2014 Nov 3;10(11):756. doi: 10.15252/msb.20145422 (PMC4299599; doi:10.15252/msb.20145422)
Supplement: Supplementary file 6 — Supplementary Figure S6 [file msb0010-0756-sd6.pdf]

Figure S6

Boeke et al. 2014

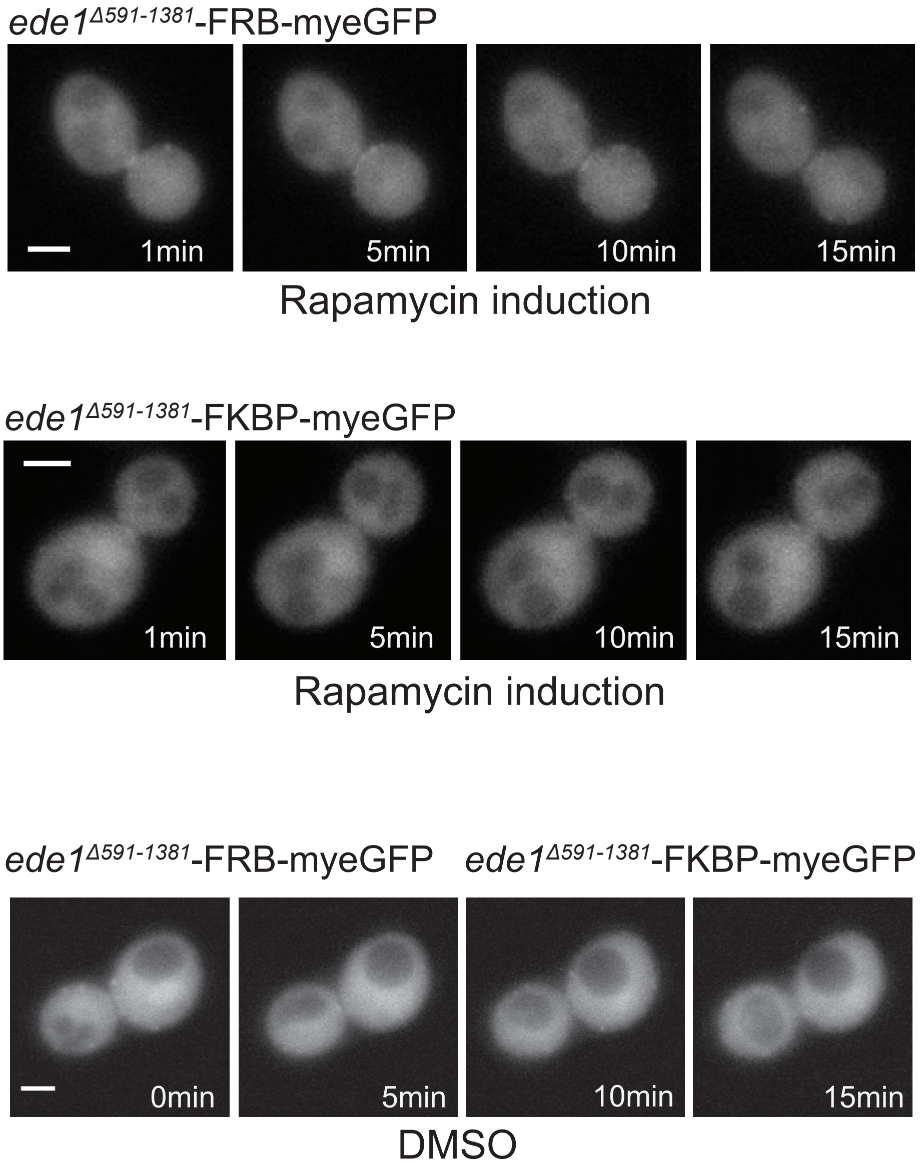

Figure S6. Time lapse microscopy of haploid *ede1*$_{\Delta 591\text{-}1381}$-FRB-myeGFP and *ede1*$_{\Delta 591\text{-}1381}$-FKBP-myeGFP cells after rapamycin treatment and of a diploid *ede1*$_{\Delta 591\text{-}1381}$-FRBmyeGFP/ *ede1*$_{\Delta 591\text{-}1381}$-FKBP-myeGFP after addition of DMSO as a control. Scale bar is 2 µm.
