# Supplementary material for: Quantification of cytosolic interactions identifies Ede1 oligomers as key organizers of endocytosis
Source: Mol Syst Biol. 2014 Nov 3;10(11):756. doi: 10.15252/msb.20145422 (PMC4299599; doi:10.15252/msb.20145422)
Supplement: Supplementary file 7 — Supplementary Figure S7 [file msb0010-0756-sd7.pdf]

Figure S7

Boeke et al. 2014

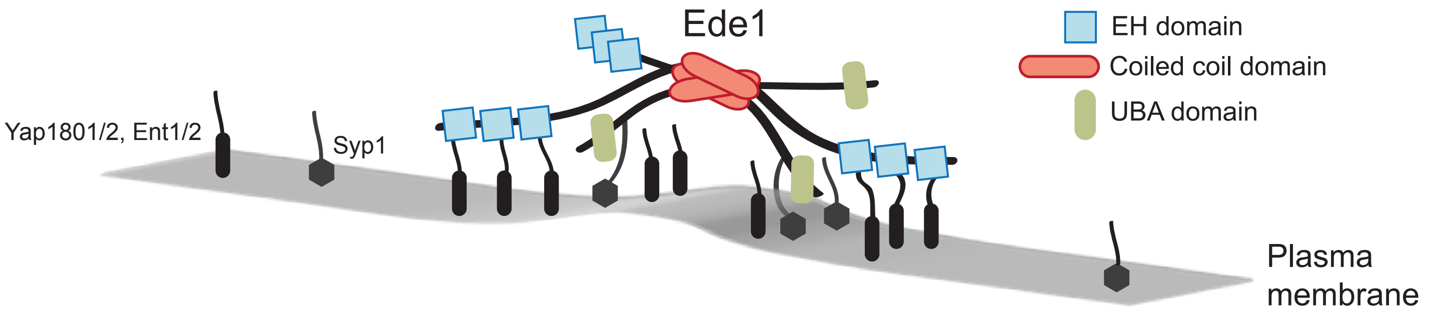

Figure S7. Model of Ede1 and its interaction with endocytic adaptors. Oligomerization of Ede1 through its coiled coil domain and binding of Ede1 to endocytic adaptors locally increases the concentration of adaptors at the endocytic site.
