# Supplementary material for: Quantification of cytosolic interactions identifies Ede1 oligomers as key organizers of endocytosis
Source: Mol Syst Biol. 2014 Nov 3;10(11):756. doi: 10.15252/msb.20145422 (PMC4299599; doi:10.15252/msb.20145422)
Supplement: Supplementary file 8 — Supplementary Tables [file msb0010-0756-sd8.pdf]

Table S1 - Overview of FCS data presented in this study (corresponding to Fig.1)

	Number of measurements	Particle concentration		Diffusion coefficient	
	N	nM	SD	µm^2/sec	SD
**1myEGFP**	53	1103	376	8,72	3,66
**3myEGFP**	30	1173	272	5,72	1,21
**Abp1 3myEGFP**	5	1206	403	2,57	0,47
**Apl1 1myEGFP**	24	33	19	1,67	0,46
**Apl3 3myEGFP**	33	47	19	1,28	0,63
**Apm4 3myEGFP**	12	32	13	1,69	0,47
**Aps2 3myEGFP**	15	88	20	3,84	1,40
**Arc18 1myEGFP**	15	368	65	4,80	1,27
**Ark1 3myEGFP**	10	16	19	2,60	1,49
**Art3 3myEGFP**	14	34	10	1,70	0,39
**Bbc1 3myEGFP**	10	78	18	1,34	0,35
**Bzz1 3myEGFP**	22	100	16	1,90	0,34
**Cap1 3myeGFP**	8	406	133	3,14	0,88
**Cap2 3myEGFP**	10	420	120	3,60	0,90
**Chc1 3myEGFP**	5	363	175	0,96	0,13
**Crn1 3myEGFP**	15	108	27	0,99	0,14
**Ede1-3myGFP**	35	138	56	0,64	0,15
**End3-3myEGFP**	21	87	30	2,42	0,96
**Ent1 3myEGFP**	16	171	28	2,96	0,62
**Ent2 3myEGFP**	17	183	40	2,60	0,73
**Inp51 3myEGFP**	7	24	7	1,78	0,47
**Inp52 3myEGFP**	7	47	20	0,81	0,17
**Las17 1myEGFP**	28	82	24	2,01	0,51
**Lsb3 3myEGFP**	13	335	98	1,78	0,64
**Myo3 3myEGFP**	21	172	61	2,14	0,45
**Myo5 3myEGFP**	13	152	30	2,58	0,39
**Pan1 1myEGFP**	52	140	45	2,19	0,71
**Rvs161 1myEGFP**	23	721	217	3,71	1,52
**Rvs167 1myEGFP**	32	354	89	3,15	1,07
**Scd5 3myEGFP**	13	31	6	2,03	0,34
**Scp1 3myEGFP**	21	163	36	4,18	0,85
**Sla1 3myEGFP**	13	427	131	1,77	0,28
**Sla2-3myEGFP**	28	116	39	1,62	0,37
**Srv2 3myEGFP**	15	162	52	0,48	0,10
**Syp1 3myEGFP**	12	45	13	1,26	0,24
**Vrp1 3myEGFP**	9	228	52	1,56	0,34
**Yap1801 3myEGFP**	20	44	17	2,43	0,82
**Yap1802 3myEGFP**	24	33	13	1,84	0,74

N denotes the number of single cell measurements. Each single cell measurement is considered to be one experiment. Particle concentration denotes the number of particles measured for 3myeGFP tagged proteins. SD represents the standard deviation.

Table S2 – Overview of previously reported interaction with the corresponding reference

		Reference
Apl1	Apl3	Luo Y1, et al. 2010. J Am Soc Mass Spectrom. 21:34-46
Ede1	Yap1802	Howard JP, et al. 2002. J Cell Biol. 157:315-26
Ede1	Syp1	Reider, A. et al. 2009. EMBO J. 28:3103–16
Ede1	Ent1	Aguilar, R.C. et al. 2003. J. Biol. Chem. 278:10737–43
Ede1	End3	Gavin A.C., et al. 2006. Nature. 440:631-6
Ede1	Sla2	Gavin A.C., et al. 2006. Nature. 440:631-6
Pan1	Yap1801	Wendland B. and Emr S.D. 1998. J Cell Biol. 141:71-84
Pan1	Yap1802	Howard J.P. et al. 2002. J Cell Biol. 1572:315-26
Pan1	Scd5	Zeng, G. et al. 2007. Mol Biol Cell. 18:4885-98
Pan1	End3	Toshima, J. et al. 2007. Mol Biol Cell. 18:658-68
Pan1	Sla2	Toshima, J. et al. 2007. Mol Biol Cell. 18:658-68
Pan1	Sla1	Tang, H.Y. et al. 2000. Mol Cell Biol. 20:12-25
Pan1	Ent1	Aguilar, R.C. et al. 2003. J Biol Chem. 278:10737-43
Sla1	Syp1	Gavin A.C., et al. 2006. Nature. 440:631-6
Sla1	Scd5	Tonikian R. et al. 2009. PLoS Biol. 7:e1000218
Sla1	End3	Tang, H.Y. et al. 2000. Mol Cell Biol. 20:12-25
Sla1	Rvs167	Stamenova, S.D. et al. 2004. J Biol Chem. 279:16017-25
Sla1	Bzz1	Gavin A.C., et al. 2006. Nature. 440:631-6
Sla1	Las17	Michelot, A. et al. 2010. Curr Biol. 20:1890-9
Sla1	Sla2	Gourlay, C.W. et al. 2003. J Cell Sci. 116:2551-64
End3	Yap1802	Howard J.P. et al. 2002. J Cell Biol. 1572:315-26
End3	Scd5	Zeng, G. et al. 2007. Mol Biol Cell. 18:4885-98
End3	Las17	Gavin A.C., et al. 2006. Nature. 440:631-6
End3	Sla2	Gavin A.C., et al. 2006. Nature. 440:631-6
Rvs167	Scd5	Tonikian R. et al. 2009. PLoS Biol. 7:e1000218
Rvs167	Rvs161	Navarro, P. et al. Biochim Biophys Acta. 1343:187-92
Rvs167	Las17	Friesen H. et al. 2003. Mol Biol Cell. 14:3027-40
Las17	Bzz1	Michelot, A. et al. 2010. Curr Biol. 20:1890-9
Las17	Bbc1	Michelot, A. et al. 2010. Curr Biol. 20:1890-9
Las17	Sla2	Gavin A.C., et al. 2006. Nature. 440:631-6
Las17	Rvs161	Michelot, A. et al. 2010. Curr Biol. 20:1890-9
Bzz1	Bbc1	Tonikian R. et al. 2009. PLoS Biol. 7:e1000218
**Self**		
Apl3		Babu, M. et al. 2012. Nature. 489:585-9*
Ede1		Wang Y. et al. 2012. Mol Biol Cell 23:3911-22
Syp1		Reider, A. et al. 2009. EMBO J. 28:3103–16
Pan1		Miliaras, N.B. et al. 2004. Traffic. 5:963-78.
Sla1		Di Pietro, S.M. et al. 2010. EMBO J. 29:1033-44
Sla2		Yang, S. et al. 1999 Mol Biol Cell. 10:2265-83
Rvs167		Lombardi, R. et al. 2001. J Biol Chem. 276:6016-22
Las17		Madania, A. et al. 1999. Mol Biol Cell. 10:3521-38.
Bbc1		Krogan, N.J. et al. 2006. Nature. 440:637-43

* The initial cut-off by Babu et al. 2012 indicated self-interaction of Apl3. However, further statistical analysis in the same study with a high confidence interaction network did not include the Apl3 self-interaction

Table S3 - Overview of FCCS data presented in this study (corresponding to Fig.2)

	Number of Measurements	Particle concentration green		Particle concentration red		Complex		
	N	nM	SD	nM	SD	nM	SD	Kdeff (nM)
Apl1-Apl1	11	39	16	26	9	No interaction		
Apl1-Apl3	16	47	23	59	23	23	13	37
Apl1-Ede1	6	37	16	96	18	No interaction		
Apl3-Apl3	13	37	13	39	13	No interaction		
Apl3-Ede1	26	71	16	115	37	No interaction		
Bbc1-Bbc1	13	57	16	73	10	19	7	115
Bbc1-Sla1	11	127	41	245	45	No interaction		
Bzz1-Bbc1	15	81	20	121	26	8	3	994
Bzz1-Sla1	17	81	16	211	54	14	10	976
Don1-Don1	17	53	12	46	12	36	10	4
Don1-Ste11	11	43	11	29	6	No interaction		
Ede1-Ede1	28	56	13	47	15	12	8	127
End3-Ede1	3	87	21	102	20	No interaction		
End3-End3	8	85	56	64	27	No interaction		
End3-Sla1	4	36	8	220	46	No interaction		
Ent1-Ede1	11	242	50	101	31	No interaction		
Ent1-Ent1	20	87	18	40	9	No interaction		
Las17-Bbc1	20	100	24	135	24	16	15	631
Las17-Bzz1	19	78	19	74	15	9	4	475
Las17-End3	7	45	25	99	31	8	6	438
Las17-Las17	2	32	9	43	2	No interaction		
Las17-Rvs161	5	54	10	311	23	No interaction		
Las17-Sla1	20	107	38	156	45	32	15	286
Las17-Sla2	7	73	41	84	23	No interaction		
Pan1-End3	34	123	27	105	25	70	18	27
Pan1-Ent1	6	110	61	158	56	No interaction		
Pan1-Pan1	17	73	17	75	21	No interaction		
Pan1-Sla1	9	140	46	163	32	No interaction		
Rvs161-Rvs161	10	197	38	160	21	No interaction		
Rvs167-Rvs161	9	211	40	274	51	196	50	6
Scd5-End3	4	39	13	102	33	No interaction		
Scd5-Pan1	16	51	15	162	39	No interaction		
Scd5-Scd5	22	23	5	40	15	No interaction		
Scd5-Sla1	5	39	11	356	45	No interaction		
Sla1-Sla1	11	361	116	132	40	No interaction		
Sla2-Ede1	11	117	24	100	24	15	3	590
Sla2-End3	3	185	54	85	24	18	6	622
Sla2-Pan1	5	97	21	109	30	14	6	539
Sla2-Sla1	2	202	21	323	0	No interaction		
Sla2-Sla2	19	83	19	58	10	19	5	133
Syp1-Ede1	15	24	6	91	27	7	3	227
Syp1-Sla1	7	16	5	128	21	No interaction		
Syp1-Syp1	13	16	4	17	8	No interaction		
Yap1801-Ede1	13	45	25	96	27	No interaction		
Yap1801-Pan1	5	49	30	164	31	No interaction		
Yap1802-Ede1	12	50	19	119	38	No interaction		
Yap1802-End3	4	34	35	106	16	No interaction		
Yap1802-Pan1	4	33	20	146	41	No interaction		

N denotes the number of single cell measurements. Each single cell measurement is considered to be one experiment. ‘Particle concentration GFP’ and ‘Particle concentration mCherry’ denote the number of particles measured for the 3myeGFP tagged proteins and 3mCherry tagged proteins, respectively. Only K_d^{eff}-values for values < 1000 nM are shown. SD represents the standard deviation.

Table S4 – Yeast strains used in this study

Strains generated for FCS/FCCS experiments in Fig. 1, Fig 2, Fig 3B and Fig. S1 were generated in the background strains BY4741 (myeGFP::natNT2 and 3myeGFP::natNT2) and Y8205 (3mCherry::hphNT1) and are not listed in this table.

Strain	Genotype	Source
**BY4741**	MATa, his3Δ1, leu2Δ0, met15Δ0, ura3Δ0	Brachmann et al. 1998
**Y8205**	MAT**α**, can1Δ ::STE2pr-HIS5, lyp1Δ::STE3pr-LEU2, his3Δ1, leu2Δ0, ura3Δ0	Tong and Boone 2007
**MKY0532**	MATa, his3200, leu2-3,112, ura3-52, lys2-801, yap1801Δ::natNT2, yap1802Δ::natNT2, apl3Δ::natNT2, EDE1-yeGFP::HIS3MX6	Kaksonen lab
**MKY0172**	MATa, his3-Δ200, leu2-3,112, ura3-52, lys2-801, EDE1-yeGFP::HIS3MX6	Kaksonen lab
**MKY2231**	MATa, ura3-52, leu2Δ1, his3Δ200, trp1Δ63, DON1-eGFP::kanMX	This study
**YCM452 (MKY2232)**	MATa, ura3-52, leu2Δ1, his3Δ200, trp1Δ63, natNT2::Pcyc-eGFP-DON1 STE11::3mCherry::hphNT1	Maeder et al. 2007
**YCM449 (MKY2233)**	MATa, ura3-52, leu2Δ1, his3Δ200, trp1Δ63, natNT2::Pcyc::eGFP-DON1::3mCherry::KanMX6	Maeder et al. 2007
**MKY2599**	MATa, his3-Δ200, leu2-3, ura3-52, lys2-801, tor1-1, fpr1::klUra3, ede1$^{\Delta 591-1381}$-FKBP-(24)-myeGFP::kanMX4	This study
**MKY2602**	MAT**α**, his3-Δ200, leu2-3, ura3-52, lys2-801, tor1-1, Fpr1::klUra, ede1$^{\Delta 591-1381}$-FRB-(24)-myeGFP::kanMX4	This study
**MKY2612**	MATa/MAT**α**, his3-Δ200/his3-1, leu2-3/leu2-0, ura3-52/ura3-0, lys2-801/LYS, EDE1-eGFP::HIS3MX6/EDE1-TAP::URA3	This study
**MKY2620**	MATa, his3-1, leu2-0, met15-0, ura3-0, Ede1-TAP::URA3	This study
**MKY2669**	MATa, his3-1, leu2-0, met15-0, ura3-0, ede1$^{\Delta 591-1391}$-myeGFP::natNT2	This study
**MKY2670**	MATa, his3-1, leu2-0, met15-0, ura3-0, ede1$^{\Delta 901-1391}$-myeGFP::natNT2	This study
**MKY2671**	MATa/ MAT**α**, can1Δ::Ste2pr-Leu2/Can1+, lyp1::/Lyp1+, his3-1/his3200, leu2-3/ leu2-0, ura3-52/ura3-0, lys2-801/Lys2+, fpr1::klUra3/ fpr1::klUra3, ede1$^{\Delta 591-1381}$-myeGFP-FRB(24)::kan/ede1$^{\Delta 591-1381}$- myeGFP-FKBP(24)::kanMX4	This study
**MKY1037**	MATa, his3-Δ200 , leu2-3,112, ura3-52, lys2-801, ADH_{pr}yeGFP-EDE1::natNT2	Kaksonen Lab
**MKY2812**	MAT**α**, his3-Δ200, leu2-3,112, ura3-52, lys2-801, tor1-1, fpr1Δ, EDE1-myeGFP::natNT2	This study
**MKY0158**	MATa, his3-Δ200, leu2-3,112, ura3-52, lys2-801, EDE1-myeGFP::kanMX4	Kaksonen lab
**MKY2814**	MATa, his3Δ1, leu2Δ0, met15Δ0, ura3Δ0, ede1$^{-\Delta EH}$-myeGFP::kanMX4	This study
**MKY2994**	MATa, his3-Δ200, leu2-3,112, ura3-52, lys2-801, tor1-1, fpr1Δ, ede1Δ:: hphNT1, SYP1-3myeGFP::natNT2	This study
**MKY2997**	MATa, his3-Δ200, leu2-3,112, ura3-52, lys2-801, tor1-1, fpr1Δ, ede1$^{\Delta CC}$, SYP1-3myeGFP::natNT2	This study
**MKY2819**	MATa, his3Δ1, leu2Δ0, met15Δ0, ura3Δ0, $EDE1_{pr}$1myeGFP::natNT2	This study
**MKY2820**	MATa, his3Δ1, leu2Δ0, met15Δ0, ura3Δ0, $EDE1_{pr}$3myeGFP::natNT2	This study
**MKY2931**	MATa/ MAT**α**, can1Δ ::STE2pr-HIS5/CAN, lyp1Δ::STE3pr-LEU2/LYP, his3Δ1, leu2Δ0, met15Δ0/MET15+, ura3Δ0, ede1$^{\Delta 591-1381}$-3myeGFP::natNT2 ede1$^{\Delta 591-1381}$-mCherry::hphNT1	This study
**MKY3012**	MATa, his3Δ1, leu2Δ0, met15Δ0, ura3Δ0, ede1^{CC}-myeGFP::kanMX4	This study

**MKY2953**	MATa/ MAT**α**, *his3-Δ200, leu2-3,ura3-52, lys2-801, tor1-1, fpr1Δ, ede1$^{\Delta CC}$ -mCherry-FKBP(24)::natNT2/ede1$^{\Delta CC}$ -mCherry-FRB(24)::hphNT1, SYP1-3myeGFP::natNT2*	This study
**MKY2823**	MATa/ MAT**α**, *his3-Δ200, leu2-3, ura3-52, lys2-801, tor1-1, fpr1Δ:: klUra3, ede1$^{\Delta 591}$ -myeGFP-FKBP(24)::kanMX4/ede1$^{\Delta 591}$-myeGFP-FRB(24)::kanMX4, SLA1-mCherry::hphNT1*	This study

Table S5 - Plasmids used in this study

Name	**Tag**	**Marker**	**Origin**
pST70 (pMK0051)	3mCherry(dcu)	*hphNT1*	This study
pST72 (pMK0053)	3myeGFP(dcu)	*natNT2*	This study
pMK0075	1myEGFP	*natNT2*	This study
pMaM57	1mCherry	*hphNT1*	This study
pGSKU-1	-	*kanMX4/URA*	Storici and Resnick, 2006
pMK0070	myEGFP-FRB(24)	*kanMX4*	Oriol Gallego (Gallego et al. 2013)
pMK0074	myEGFP-FKBP(24)	*kanMX4*	Oriol Gallego (Gallego et al. 2013)
pBS1539	TAP	*URA*	Puig et al. 2001
pKS133	-	*hphNT1*	Janke et al. (2004)
pKS134	-	*natNT2*	Janke et al. (2004)
pFA6-kanMX	-	*kanMX4*	Knop lab
pYM–N9	ADH_{pr} - yeGFP	*natNT2*	Janke et al. (2004)

dcu = different codon usage (for each repeat of the florescent protein)

Supplementary References

Brachmann CB, Davies A, Cost GJ, Caputo E, Li J, Hieter P, Boeke JD (1998) Designer deletion strains derived from Saccharomyces cerevisiae S288C: a useful set of strains and plasmids for PCR-mediated gene disruption and other applications. *Yeast* **14**:115-32

Storici F, Resnick MA (2006) The delitto perfetto approach to in vivo site-directed mutagenesis and chromosome rearrangements with synthetic oligonucleotides in yeast. Methods Enzymol **409**:329-45

Gallego O, Specht T, Brach T, Kumar A, Gavin AC, Kaksonen M (2013) Detection and characterization of protein interactions in vivo by a simple live-cell imaging method. PLoS One **8**:e62195

Puig O, Caspary F, Rigaut G, Rutz B, Bouveret E, Bragado-Nilsson E, Wilm M, Séraphin B (2001) The tandem affinity purification (TAP) method: a general procedure of protein complex purification. Methods **24**:218-29
